# Community/patient group champion team retrospective look at engage for equity PLUS: Results from a post-intervention champion team focus group

**DOI:** 10.1017/cts.2025.10092

**Published:** 2025-07-07

**Authors:** Michael Muhammad, Paige Castro-Reyes, Marty Chakoian, Ysabel Duron, Starla Gay, Howard Grant, Bridgette Hempstead, LaShawn Hoffman, Diane Mapes

**Affiliations:** 1 Community Consultant, University of New Mexico, Albuquerqu, NM, USA; 2 Deputy Director, Community-Campus Partnerships for Health (CCPH), Engage for Equity 2 National Board, Raleigh, NC, USA; 3 Us TOO International Prostate Cancer Education and Support Network, Patient Advocate Member of Champion Team, Fred Hutch/University of Washington Cancer Consortium, Seattle, WA, USA; 4 Founder and Executive Director, The Latino Cancer Institute, Patient Advocate Member of Champion Team, Stanford University, Stanford, CA, USA; 5 Founder, Black Ladies Advocating for Cancer Care, Patient Advocate Member of Champion Team, Stanford University, Stanford, CA, USA; 6 Member, Community Coalition Board, Morehouse School of Medicine, President and CEO of Fulton Atlanta Community Action Authority, Atlanta, GA, USA; 7 Founder and Director, Cierra Sisters, Patient Advocate Member of Champion Team, Fred Hutch/University of Washington Cancer Consortium, Seattle, WA, USA; 8 Chair, Community Coalition Board, Prevention Research Center, Principal, Hoffman and Associates, Morehouse School of Medicine, Atlanta, GA, USA; 9 Founder, Lobular Breast Cancer Alliance, Patient Advocate Member of Champion Team, Fred Hutch/University of Washington Cancer Consortium, Seattle, WA, USA

**Keywords:** Community-based participatory research, community-engaged research, patient-engaged research, institutional transformation, Academic Health Centers

## Abstract

Community/patient voice has long been stifled in favor of the priorities of powerful health organizations that set the agenda for institutional practices and policies shaping health equity research. Academic Health Centers (AHC) and Clinical Translational Science Centers (CTSC) promote missions that are often unaligned with the realities of community and patient experiences when interacting with researchers and representatives from these institutions. Implementation science has increasingly adopted collaborative and participatory approaches to the design and implementation of health interventions co-created with community/patient group members as equal participants within community-academic partnerships. Community-based participatory research/community-engaged research are widely recognized as approaches to health intervention research that offers the potential for community-patient voice to be heard when the principles of authentic participatory research are adhered to throughout all aspects of the project. For AHC’s and CTSC’s to be fully engaged, the populations they serve must have access to institutional leadership and influence over decision-making about the organizational resources allocated to community/patient groups beyond efforts to cultivate a positive public image. The E2 community/patient champion team focus groups provide unique perspectives on how equitable institutional transformation can be accomplished in a retrospective assessment of the E2 PLUS Intervention.

## Introduction

The legacies of systemic oppression and marginalization in the U.S. (i.e., colonization, chattel slavery, and sexism) have led to deeply entrenched structural inequities that in turn, shape the social and political determinates of health equity. Implementation science has evolved over the years to acknowledge that power-sharing and privileging of community-voice are fundamental tenants of collaborative approaches to conducting community-academic research [1–3]. Community-engaged research (CEnR) and community-based participatory research (CBPR) are essential to advancing authentic engagement of community members in evidence-based strategies and enhancing infrastructures to further research for health equity. Increase in the prevalence of Community Engagement Cores (CECs) in Clinical Translation Science Centers have led to the broad adoption of CEnR/CBPR principles in patient/community-academic partnerships [4].

In 2021, UNM was awarded a PCORI Science of Engagement Award to test the feasibility of its Engage for Equity Plus (E2 PLUS) intervention. UNM proposed to test feasibility of implementing the UNM E2 PLUS intervention with three national institutional partners: 1) Morehouse School of Medicine (working with the Prevention Research Center); 2) Fred Hutchinson/University of Washington Cancer Consortium (working with their Office of Community Outreach and Engagement); and 3) Stanford School of Medicine and Stanford Cancer Institute (working with the CTSA Office of Community Engagement and other Departmental offices). The Engage for Equity (E2) study (2015–2022) identified partnering practices, metrics, training and tools that support community/academic partnerships at the project level [5,6]. E2 PLUS retained the original E2 intervention components with the addition of champion teams comprised of 6–8 individuals representing two subgroups: academics (e.g., community engagement core directors, investigators, and staff), and patients, patient advocates, and community members; preparing them to be facilitative leaders working collectively towards institutional change.^4(p3),7^ Preliminary data from the E2 PLUS study demonstrated evidence that enhancing facilitative leadership among champion team members is an effective institutional change strategy, where feasible institutional change targets are identified; working groups are developed to address targets; and qualitative and quantitative data presentations enhance leadership buy-in.

UNM proposed to scale up the evidence-based E2 intervention working with partnerships, to work with champion teams at each institutional site in order to identify and target policies, practices, and norms that would strengthen equity-based patient and community engaged research (P/CEnR) [8]. The E2 PLUS intervention aims to: 1) conduct institutional assessments of facilitators and barriers to equity-based P/CEnR through leader interviews, focus groups, and surveys; 2) create champion teams of 6–8 members including faculty, staff and patient advocates/community members; 3) provide multiple workshops to ∼ 25 additional participants using E2 Tools, dialogue, and collective reflection; 4) provide ongoing coaching to Champion Teams; and 5) create a multi-institution community of practice to enhance co-learning and uptake of practices and policies of equity-based P/CEnR. The E2 PLUS intervention is guided by a CBPR model recognizing community knowledge, lived-experience, power inequity, and nuanced understanding of cultural factors that contextualize health equity research are vital to the sustainability and impact of local health interventions.^3(p3),8(p4),9^ E2 PLUS intentionally created champion teams that include community/patient group members as equal partners to ensure that community expertise and perspectives about transforming Academic Health Centers and institutions address the concerns and priorities of affected communities.^2(p3),10^ To enhance the usefulness of the E2 PLUS for non-academic partners we invited community/patient group champion team members from three institutions Fred Hutch/UW Cancer Center, Stanford Medical Center and Morehouse School of Medicine having completed the two-year E2 PLUS intervention from 2021–2023 (e.g., workshops, working groups, training, and coaching sessions) to provide a retrospective assessment of champion team coaching, E2 Plus tools, and persistent barriers to institutional transformation for enhancing community/patient group engagement.

As part of the E2 PLUS intervention champion teams met separately (i.e., community/patient groups and academic) and were afforded the opportunity to provide feedback for improving workshops, training, and coaching sessions. During routine research team debriefs, the community consultant who led several community/patient groups only workshops noted that the absence of academic partners created an atmosphere for openness and trust in sharing perspectives about E2 PLUS and institutional culture, policies and practices that may prove resistant to change. Participants appeared to be more willing to offer critical views of E2 and the responsiveness of academic champion teams and their institutional leadership to change in the closed setting. This perspective surfaced during the community/patient champion team focus group. For example, one participant was willing to be labeled a “jerk” for suggesting that entrenched power inequities in community engagement may persist due to the characterization of community members by some in academia as “ignorant,” “old,” “poor” or “people of color.” Western science (especially in translational, implementation, and intervention research) is grounded in a positivism and rigor that privileges advanced technical training and degrees earned through formal education. Institutional power is conferred to university trained researchers who risk being socialized into an academic elitism in the valuing of academic knowledge exclusively. Authentic community/patient engagement recognizes the benefit and value of knowledge co-creation in community/patient-academic research partnerships. Co-produced knowledge includes respect for diversity in life experiences, cultural values, and different ways of interpreting information that are critical for positive health outcomes. Devaluing non-academic knowledge and ways of knowing effectively maintains power inequity and undermines trust within community/patient-academic research partnerships [11]. In preparation for a new funding opportunity that would test a scaled-up version of E2 PLUS the UNM research team decided to solicit the perspectives of the community/patient group champion team with a final focus group. The UNM research team was in the process of developing a proposal for a PCORI Science of Engagement award and wanted to acquire additional insights from community/patient group champion team members for improving adaptations to the new E2 PLUS intervention.

## Methods

Eight community/patient group champion team members and one member of our national community-engagement organization agreed to participate in a focus group (without the presence of academic champion team members). The community consultant recruited the participants through an email invitation and developed the focus group guide in collaboration with the UNM E2 Plus team. The focus group was conducted using the Zoom platform approximately 9 months after the conclusion of the E2 in February 2024. The interview guide was sent to all participants prior to the focus group session. Participants were asked to respond to semi-structured questions on three components of the E2 PLUS intervention: champion team coaching, uptake of E2 tools and the persistence of institutional barriers for community/patient engagement. Due to lack of time, the two remaining questions about the champion team’s capacity and outcomes were not asked. The one-hour session was recorded and auto transcribed using the Zoom feature. The community consultant reviewed the transcript for accurateness and categorized responses for each question on an excel spreadsheet using a combination of thematic analysis and modified grounded theory [12,13]. As participants of the E2 PLUS intervention focus group members were not compensated for their time.

## Results

Tables 1- 4a report selected themes, concepts, and quotes identified by participant alpha/numeric codes (A1 – H8) assigned during coding for confidentiality. The data are organized by focus group question (abbreviated in tables for space). In this brief report we summarize key insights on two critical areas: 1) coaching aspects of the E2 intervention and 2) institutional barriers to community and patient engagement. The full results of the qualitative data are reported in a separate paper submitted for this special themed issue [14]. In response to the question on champion team coaching a discussion on the use of the term “coaching” within the context of the E2 PLUS intervention highlighted the nuanced understanding of coaching based on experience level and the importance of peer-level conversations in the collaborative process. One perspective shared by a well-respected leader in community-engagement and patient advocacy emphasized a disconnect between the intended coaching activities and her experience of the project. She highlights a perceived one-sidedness in the process, focusing on institutional change without clear benefits for the community. *Well, what’s in it for us, the community? We keep on making you guys better. What are you doing?* [E5] This reflection raises questions about the communication of the project’s goals and the use of the term “coaching,” suggesting a gap between the project’s implementation and its perception among participants. Another participant with a background in CBPR and community-engagement noted the value of the coaching process in engaging community members and highlighting the challenge of ensuring community partners are included in significant conversations. He notes that other community partners not participating in the E2 workshops and training could have gained from the rich dialogue about institutional transformation for community engagement and suggests a critical reflection on how to better integrate other community partners in coaching strategies for institutional change. *I firmly believe that the community partners missed this opportunity of having some of these higher-level conversations that took place on our coaching strategies… I was the only community person that was there*. [H8] His reflections capture the appreciation for the coaching’s impact on community engagement but also critically addresses the limitations of the availability of “coaching” and the process, particularly regarding how to extend the reach of E2 information and resources to community partners not able to take part in the periodic sessions and the inclusion of community partners in meaningful conversations and strategies beyond the project. Participants also highlighted the different perceptions and impacts of coaching for community and patient partners. The exchange of views over the complexity of E2 coaching dynamics raised several questions about who was actually being coached and in what ways did the E2 activities facilitate bi-directional learning, suggesting that while some more experienced partners may have viewed coaching in terms of a mutual sharing of knowledge (from an extensive background in community/patient engagement), other community partners found value in the opportunities for information exchange leading them to reflect on how the meaningful conversations could be disseminated more broadly.

**Table 1.** Champion team community/patient focus group selected key themes, concepts, and quotes

Focus Group Question 1: General assessment of the coaching to champion teams	
**Theme:** Coaching & Capacity Building	**Concept:** Coaching Effectiveness & Community Inclusion
**Quote:**	
I appreciated the coaching. Because what it forced me to do as part of our champions team was to reach out to our community members to ensure that I was sharing and that they were aware of the work that was taking place… I know that we were looking to do institutional change, right? And that we wanted to ensure that the academic partners were there. But I firmly believe that the community partners missed this opportunity of having some of these higher-level conversations that took place on our coaching strategies…I was the only community person that was there. **H8**	
**Theme:** Misplaced Concept of Coaching	**Concept:** Misplaced Concept of Coaching
**Quote:**	
What’s misplaced here for me is the whole word coach. I didn’t know there was coaching going on. I thought we were having these conversations between community groups and academics about what the barriers are to a successful meeting of the minds and moving forward on change. But it seems like it’s really one-sided. How do we do institutional change? What are those barriers to institutional change that make us difficult to work with you, the community? Until we started able to have the conversation about, well, what’s in it for us, the community? We keep on making you guys better. What are you doing? You know, and so there was only a partial change… **E5**	
**Theme:** Coaching Dynamics	**Concept:** Coaching Dynamics
**Quote:**	
I think for maybe some of the institutional partners it was like a coaching of these are things that you need to be considering and implementing in your institutional practices. But then for community partners…I think the benefit was a connecting in a space to talk more clearly and specifically about the experience of community partners through the institutional dynamics that were being evaluated. And so I think … there was some dynamics within the partnership that may have shifted throughout this engagement. There was also mentoring and feedback and expertise that the community partners were functioning as well in the dynamic with the institutional partners. **G7**	
**Theme:** Coaching Dynamics	**Concept:** Academic-Community Language Dynamics
**Quote:**	
[T]here is a tendency for the academics at the at the table to get on a little academic. High Horse and that’s not to say that anyone on my team did that but academics kind of lead in a certain way, right? And always reminding to try to bring it back because there are community partners at the table and we do not necessarily speak the same language. And if you want us to speak the same language, we’re gonna have to determine between us what that language is going to be. **B2**	

*N* = 8; Focus Group Participant IDs = H8,E5, G7, and B2.

**Table 2.** Champion team community/patient focus group selected key themes, concepts, and quotes

Focus Group Question 2: Uptake of E2 Tools and Best Practices	
**Theme:** Implementation and Impact	**Concept:** Limited Progress
**Quote:**	
I think that the academic members of the champion team were able to [it] put on the radar of leadership. [T]hey were able to, elevate it, so there [was] the task force that was created to talk about more diversity in clinical trial. I think the most important thing when they put it into a proposed budget to fund patient engagement. I think those contributions by the academic members of the champion team are really important. Now. I haven’t heard much on what happened since the budget was approved. And the leadership at [Institution] stuck the office of patient engagement into an already existing office of patient experience. There’s been no movement that I know of since that point. **A1**	
**Theme:** Implementation and Impact	**Concept:** Real-life Impact & Advocacy
**Quote:**	
It occurred to me that we were not adequately representing patients in prostate cancer research. And based on the things that I heard from the other folks at [Institution], it seemed like [Institution] might be open to more patient involvement. And so. One of the things that I suggested is that the patient advocates around prostate cancer actually get involved and embedded in the research projects as patient advocates. On a sort of a real-time basis in that research and that we get involved in prioritizing research project requests for funding. And both of those. Ideas were approved by the folks at [Institution] in the Prostate Cancer Research community. And we are now doing those things. So I feel really good about that. That advocates are much more involved in a meaningful way in the research that’s going on. **C3**	
**Theme:** Communication and Accountability	**Concept:** Communication and Accountability
**Quote:**	
[P]art of the problem remains the same. Communication going out coming back to the to the institution and saying, okay guys, we did this for 2 years. We had all these proposals for implementation and change. Now we can report back to you that we accomplished this, this, and this. And so to me it’s kind of knowing how much my colleagues at [Institution] and others are super busy themselves going on to the next best thing. Which is part of the whole problem of academia. It’s always moving on to the next best thing to write the next grant to finish off or to keep on top of stuff to accomplish all things that unfortunately academics need to do in order to be part of that life. And so they talk about community engagement and then put aside some of the most important critical things which [is] communication both ways. **E5**	

*N* = 8; Focus Group Participant IDs = A1,C3, and E5.

**Table 3.** Champion team community/patient focus group selected key themes, concepts, and quotes

Focus Group Question 3: Academic Advocacy for Patient/Community Groups	
**Theme:** Change In Institutional Approach to Community Engagement	**Concept:** Change In Institutional Approach to Community Engagement
**Quote:**	
I will tell you that I started to see the change in the academic partners as they start to think about how to engage community… community patients and their work so much so that I can tell you that I think it was part of the shift that was needed as [Institution] started to write its new strategic plan to incorporate community engagement as a pillar that wasn’t there before. So they’ve always said we do community engagement but it wasn’t in the strategic plan as a core pillar of its work and it’s there today as a core pillar of the work. So that everyone at the institution understands how important this is and not just a specific center or specific people because this is their work. **H8**	
**Theme:** Change In Institutional Approach to Community Engagement	**Concept:** Change In Clinician View of Community Engagement
**Quote:**	
And that’s a huge change even for [Institution] and specifically I can say some of the other clinicians that say we just do basic science [bench?] stuff, right? We do not do that community “feely” stuff. I’ve started to see the change in all of that saying, no, the basic scientists also should be doing community engaged research for that includes both community and patients right patient advocates because of what we’re attempting to do and how we’re trying to change the world and improve health equity. **H8**	
**Theme:** Challenges In DEI Initiatives	**Concept:** Challenges In DEI Initiatives
**Quote:**	
I do not work directly with patients anymore [as] I used to…I now work at basically a state and national level and it is actually to address these larger questions when we keep talking about DEI…We just put a new little name on it and we’re suddenly feeling it again. But already I’m seeing pushback. In communities of color about this DEI stuff, yeah, everybody is co-opting it, but what really are you showing that this may change within your institutions? You’re cutting back people, you’re, you know, suddenly the DEI office [has] gone away or it’s buried under something else. **E5**	
**Theme:** Community Engagement Challenges	**Concept:** Listening to the Community
**Quote:**	
You know, and I think that that’s all because we’re the experts and we know it all and you know, so I think that the step [Institution] is taking has taken rather is it opens the door now to really you know, looking introspectively at how I’m listening to the community. You know, as opposed to just showing up, you know, your presence at the table doesn’t necessarily mean that you know you’re going to be heard. You know, but I wanna listen to you so I can understand so we can take action together versus me coming up with well, we’ve done that and we’re doing that and we plan to do that and that’s listening to respond. **F6**	

*N* = 8; Focus Group Participant IDs = H8,E5, and F6.

**Table 4.** Champion team community/patient focus group selected key themes, concepts, and quotes

Focus Group Question 4: Barriers To Institutional Change in Patient & Community Engagement	
**Theme:** Institutional Barriers	**Concept:** Institutional Requirements as Barriers
**Quote:**	
[W]e list community issues problems right and then you have institutions that are supposed to be there whether it’s academia or any other institution that’s supposed to be there to help, but you have all of these requirements that really are barriers, you know, moving forward. So all you’re doing all it is… is lip service. And one of the things that I’m proud of [Institute] in terms of, you know, the step towards incorporating engagement into the strategic plan is it gives us an opportunity to start chipping away at one major barrier and that barrier is oftentimes we go out into communities and we listen to respond but we do not listen to understand. **F6**	
**Theme:** Institutional Commitment for Community Engagement	**Concept:** Lack of Follow-Up
**Quote:**	
I worry with the progress at instituting some kind of office for patient engagement that it was just a lip service. I worry that it’s that they just provided for funds, they stuck it under. A group, an office that already existed. And there it’s gonna die and it’s just gonna be buried. Because the people who are running that office. They’re not involved in research. They are the people that patients go to complain when something goes wrong. So they are a de-escalation. Basically, they’re the patient experience team and they stuck the engagement under those people. So I worry that it’s gonna die there that it’s not gonna blossom and flourish. **A1**	
**Theme:** Institutional And Academic Power-Sharing	**Concept:** Power Inequity
**Quote:**	
Yeah, well, I mean, you’ve touched on 2 really important things, [A1]. And one is I think a barrier, a really significant barrier that we do not pay enough attention to, community engagement is the power barrier. Because if you are really going to share decision making with people in the community who are impacted by those decisions. That means you’re giving up some of your power to make those decisions. And that’s very, very hard for people to do. And I’m just going to be a little bit of a jerk here. And say that’s especially hard for people in academia or people with, you know, advanced credentials to do because they’ve invested a lot of their time and effort and money into getting those credentials and why should they share power with you know ignorant people like me who are just you know old people with cancer right? Or poor people or people of color or whatever, definition you want to use. We all can identify underrepresented and underserved communities and sharing that power is hard and that is a real barrier. **C3**	
**Focus Group Question 4:** Barriers To Institutional Change in Patient & Community Engagement	
**Theme:** Funding & Resource Allocation	**Concept:** Community Investment
**Quote:**	
The other barrier that you brought up is money. And, you know, frankly, I do not care how much money, [Institute] pays for some, you know, internal office that creates checklists and examines itself…outreach to the community is expensive because you need to go out into the community, and you need to have the people that are going into the community…trained to listen and to hear and to create solutions to what the community is observing. And so that’s an important investment. And I do not know whether that investment is being made at [Institutions] or anywhere else because it’s not visible to me. **C3**	
**Theme:** Funding & Resource Allocation	**Concept:** Financial support for Patient Engagement
**Quote:**	
But with regard to Working with patients. Who want to sit down at the table and be, you know. And work with researchers as an equal, we’re not seeing money spent on that. That’s the patient engagement piece of the puzzle that we need. That is where there is this sticking point. **A1**	
**Theme:** Community Mistrust and History of Institutional Commitments	**Concept:** Community Mistrust and History of Institutional Commitments
**Quote:**	
You know, another barrier I find is, when we go out into community, we get frustrated because we’re not accepted and that, there’s a lot of doubt and mistrust. I think also understanding the history of communities. Understanding the promises made and the promises not kept that occurred in communities that caused that distrust. I think us doing just a little bit more homework. Maybe even having individuals in the community that we can talk to… to find out what the history is what’s the background, what promises were made what promises were not kept. When we finally do go out as a team, the team now understands the dynamics in the room. You know, they say, know, your room. Well, we understand now that dynamics in the room and our approach. Is again going back to what we said earlier. It’s not as academic but is more empathetic to what got you here in the first place. So, I do think just understanding the background of the communities that we’re dealing with. Even as an [Institution] right smack in the middle of [a racialized] community, sometimes we lose sight of that because we’re behind those walls. **F6**	

*N* = 8; Focus Group Participant IDs = F6, A1, and C3.

In an observation about the monthly coaching meetings another participant with experience in patient engagement and advocacy reflected on the nature of the sessions, also questioning its categorization as “coaching” and highlighted the challenges of equity and accessibility for community partners not embedded in academic contexts. Somewhat unsure of what specific activities represented coaching; she called attention to the normal conventions that researchers use in communicating specialized knowledge formulated during academic training. The onus has always been on the community/patient partners to build their capacity to comprehend academic terms and jargon without a similar investment in learning how to communicate effectively to broader audiences. *There are community partners at the table, and we do not necessarily speak the same language. And if you want us to speak the same language, we’re gonna have to determine between us what that language is going to be.* [B2] She underscored the importance of clear, plain communication to bridge the gap between academic language and community understanding, advocating for a more inclusive approach to collaboration. But adds that the agreed upon forms of community-academic communication must be negotiated between community/patient and academic partners highlighting an often-unseen form for maintaining power inequity in community/patient-academic research partnerships. Community/patient group members that routinely work in partnership with academics recognize when they are being excluded from the conversation when academics employ technical jargon, acronyms (e.g., CBPR, NIH, CTSA), and disciplinary conventions without ensuring that all partners understand the language used. One’s positionality within a knowledge production hierarchy is often communicated through language. University researchers may tacitly reinforce the misperception that community/patient knowledge and language is less valuable than academic knowledge to maintain existing power inequities. Participatory and collaborative approaches to conducting research with community/patient partners require power-sharing as a fundamental principle of authentic engagement. Negotiating how the partnership will communicate and disseminate information is often a first step early in the partnership formation phase.

The rich dialogue about the dynamics and meaning of the term coaching led to general observations about the often-overlooked inaccessibility of academic language for community partners. Insights such as these underscore the challenges faced by patient and community partners who are not deeply embedded in the academic or scientific community, particularly in terms of understanding and using specialized language and acronyms. A perspective shared by a focus group member on the difficulties of navigating highly technical academic research language suggests that there is a need for more inclusive and accessible communication practices within collaborative projects. She draws from her own experiences as a patient advocate within an academic research institution and her efforts to bridge the communication gap for the benefit of patient advocacy. A similar observation about language accessibility was made by a different participant with several years as an organizational leader and long-time member of CBPR partnerships. *It was reminding me of some things that maybe I need to tweak. In my approach, you know, towards engaging the community. But again, just looking at language and then trying to break down the barrier, the psychological barrier that may exist between the community and the institutions*. [F6] He emphasizes the need for culturally appropriate communication practices that consider how existing power inequities, shape the language preferences, and cultural sensitivities of the community. He also reflects on his own approach to community engagement and acknowledges the value of self-reflection and continuous effort to build meaningful relationships that enhance trust between institutions and the community. In other words, institutional as well as individual trustworthiness and respect for community/patient members is conveyed through the intentional use of clear and straightforward language which often requires additional effort on the part of academics to simplify for the average person. The use of accessible, culturally appropriate language may be overlooked as a necessary feature in establishing trust with marginalized populations. It is difficult to convey genuine humility when entering communities as an outsider (e.g. academic researcher) while using specialized technical language or titles that reinforce differences in levels of formalized education. Academic researchers seeking to co-create equitable community/patient-academic research partnerships will often divest themselves from symbols of power and authority by meeting with partners in a community setting (i.e., a community center of power) and preferring not to be identified by their academic title (e.g., Dr). These approaches help to reduce power differentials between partners and community members. The acquisition and use of culturally appropriate language demonstrates respect for cultural values, community history, and an appreciation for diversity of experiences.

### Institutional barriers to community and patient engagement

Another key topic of the discussion centered around a question about the presence of institutional barriers for community/patient engagement. One powerful testimony (a community partner with a background in CBPR and community-engagement) illustrated how inconsistent dissemination of institutional policies can impede the work of building trust with communities, even after the institution has completed the rigorous process of securing grant funding.

*We just got this wonderful grant to do work with formerly incarcerated people and part of it says that we want to have peer champions, people that have been previously incarcerated, but … they cannot be hired by the institution because the institution says, felons cannot be hired. But what the hell are we doing? And why is the policy there and why did we go after it in the first place?* [H8]

He shared his frustration caused by policy barriers that should have been addressed during the grant application process. This example too often typifies how siloes form within organizations that are somewhat isolated from the realities of community/patient engagement work undermining institutional trustworthiness when communities are effectively denied access to institutional resources after agreeing to become a partner.

A colleague from the same institution concurred underscoring the existence of institutional barriers that hinder effective community engagement efforts. He highlighted the discrepancy between the professed intent to help communities and the actual requirements that pose significant barriers, emphasizing the need to move beyond lip service towards genuine understanding and action. Although he believes that the incorporation of community engagement into his institution’s strategic plan is an *opportunity to start chipping away at one major barrier*. He stated that, *oftentimes we go out into communities, and we listen to respond but we do not listen to understand*. [F6] His sentiment highlights the importance of cultural humility and a genuine commitment towards engaging communities as equals, demonstrated by active listening. A process that conveys the view that community knowledge and experiences are valued not simply for use as data, but the people themselves are a valuable resource that institutions should rely upon as partners in health equity and community/patient advocacy work. A participant at a different institution (a leader in patient advocacy and engagement) suggested that one important barrier within academic institutions is power inequity. Academic researchers and the institutions they are affiliated with are often reluctant to share decision-making power with community members and patient groups.

*If you are really going to share decision making with people in the community who are impacted by those decisions. That means you’re giving up some of your power to make those decisions. And that’s very, very hard for people to do…especially hard for people in academia or people with advanced credentials to do because they’ve invested a lot of their time and effort and money into getting those credentials and why should they share power with you know ignorant people like me…*[C3]

He points out that budget allocation reflects genuine commitment to specific initiatives and demonstrates institutional priorities. He suggests that speeches alone are insufficient without corresponding budgetary investments, indicating the significance of financial resources in addressing barriers to community engagement within research institutions. Power-sharing is central to participatory and collaborative approaches to CEnR. This theme emerged throughout the focus group discussions with respect to critical components for transforming the nation’s academic research centers to be more responsive to the needs and priorities of patients and community members.

In agreement, a participant with experience in patient advocacy and engagement described the disparity in funding allocation between community outreach initiatives and patient engagement efforts of her institutional partner. While the institution invests in community outreach programs, such as health fairs and events, funding for patient engagement initiatives appears to be lacking. *[W]ith regard to working with patients who want to sit down at the table and work with researchers as an equal, we’re not seeing money spent on that. That’s the patient engagement piece of the puzzle that we need. That is where there is this sticking point.* [A1] This discrepancy underlines a significant barrier to achieving equitable collaboration between researchers and patients, hindering progress in genuine sustainable patient engagement within the institution.

Institutions may publicize their desire for diversity and inclusion, but this often translates into recruitment efforts to include greater numbers of underrepresented minoritized groups in clinical trials. For some of the focus group participants, community mistrust of academic institutions is still a factor for community engagement. One focus group member was rather forthcoming about some of the challenges community partners undergo working in community-academic partnerships. He described the experience of being perceived by community members as untrustworthy due to his and other partners’ affiliation with academic institutions that have breached community trust in the past.

*When we go out into [the] community, we get frustrated because we’re not accepted and there’s a lot of doubt and mistrust. I think also understanding the history of communities. You know, understanding the promises made and the promises not kept that occurred in communities that caused that distrust.* [F6] Marginalized communities continue to be wary of university outreach initiatives due to the lack of connectedness to the communities they serve. Communities of color may feel that they have value to institutions when it largely benefits the institution. When prior commitments to communities are not sustained either through change in leadership, policy initiatives, or budgetary allocations, mistrust of institutional motives for community outreach develop. Communities form a collective memory about institutions based on experiences interacting with representatives of the institution. One approach for overcoming community mistrust of institutions was shared by a patient advocate. She speaks about how her partner institution talks about community engagement but lacks sufficient follow-through to demonstrate that desire. *Why do not you just, you know, bring yourself into the community and do it? What’s needed? The community does not know how to engage with [Institution] and the community does not know what [Institution] has to offer. And the community does not even know how to approach you. And so let’s start there.* [B2] She suggests that the institution create community “training hubs” within the specific communities the institution has a focus on. She recommends that the institution develop alliances with key community partners and resource centers in the communities of interest and offer community trainings as a way of demonstrating genuine commitment for community engagement.

These reflections point to a gap in the preparation of the nation’s academic research and bio-medical workforce. Funders increasingly require that CEnR and/or CBPR guide research design and implementation when marginalized communities are involved in the proposed research. There is a need to incorporate antiracist frameworks and methods in graduate level training in CEnR and CBPR to account for the multilevel influence of racism on community trust of academic research in an effort to better prepare researchers to value a broader spectrum of knowledge creation beyond the academy. ^11(p99),15^ A greater appreciation for the knowledge and insights gained from community and patient lived-experience should translate into a respect for decisions that reflect their priorities in research as well as those of academic researchers.

## Discussion

The retrospective focus group (conducted approximately 9 months after the conclusion of the E2PLUS study) gave participants an opportunity to reflect on the usefulness of the E2 PLUS intervention and identify areas for improvement. It also provided an opportunity to re-examine institutional capacity and entrenched power inequities (i.e., privileging academic knowledge, non-inclusive communication styles, non-use of culturally appropriate language, and a lack of shared decision-making) for authentic community/patient group engagement beyond mere “lip-service [16–20].” Horowitz et al. echo this concern questioning whether academic researchers are prepared to genuinely share power with community partners. Cautioning that increased attention and funding for CBPR could potentially “lead to a surge in name only CBPR.”^21(p2639)^ The concept of champion team coaching was vigorously contested by focus group members that had decades of experience in community-engagement and patient-advocacy. The principles of co-learning and knowledge-sharing are fundamental to CBPR and collaborative approaches to implementation science[22–24]. The use of the term coaching has been retained for the new PCORI award (PCORI #33314). The UNM team will continue to investigate how different partners understand coaching on the use of E2 PLUS tools and resources. The E2 PLUS intervention routinely captures participant insights through workshop and team debriefs, the collection of post-meeting “plus-deltas” and the solicitation of participant “a-has” (i.e., spontaneously apprehended insights derived from on-the-spot reflections of E2 activities and processes). This qualitative data is often utilized by the UNM team to revise and modify components of the intervention.

One such modification came from community-patient group champion team observations about the relationship between power inequity and institutional transformation. Academic Health Centers and their CECs within CTSC’s possess enormous amounts of power that could create an organizational climate increasing community-patient group access to institutional planning and resource allocation enhancing community-patient engagement. During the course of the E2 PLUS study (2021–2023) input from community-patient group champion team members and the E2 External Governing Board led to the creation of an untested engagement method: Institutional Patient/Community Action Groups (PCAG) that will conduct outreach to build capacity beyond patient/community members currently connected to AHCs and Institutions.

The patient/community action group (PCAG) is made up of patients, advocates and community members, who together discuss work on strategies to outreach and build capacity for patient advocates and community members outside of the institution. PCAG members are recruited by members of the E2 PLUS Champion teams established at each of the institutions participating in the study. The goal of the PCAG is to expand external networks and build capacity of individuals and organizations not yet involved in research to newly participate, to recruit others to participate in research, and to gain capacity to co-lead and lead research in the future. Specific aims of the patient/community action group (PCAG) are as follows: 1) identify patient and community “non-negotiable demands” of how research should be conducted and how the institution should respond; 2) collaborate with PCAG members to develop strategies for improving patient/community involvement within the institution and in their communities; and 3) use data to engage and advocate to institutional researchers, faculty and institutional top leadership to promote patient/community leadership in research. PCAG members will participate in E2 PLUS workshops and trainings designed to enhance skills related to building patient/community capacity, strengthening leadership in research, enhancing external outreach to new partners, empowerment and patient/community advocacy.

We hypothesize that the effectiveness of E2 engagement methods will be strengthened through the collaboration of champion teams, community advisory boards and PCAG members leading to expanded community networks with increased trustworthiness in research and capacity for community/patient-centered leadership in research. The addition of the PCAG’s as a new component of E2 PLUS will be tested in the next round of PCORI funding. As communities become more proficient in the factors for conducting equitable community research, they will be better situated to hold AHCs and Institutions accountable to the communities and patients they serve.

In conclusion, focus group participants recognized the value of the E2 PLUS Intervention in several important areas. First, the opportunity to collaborate and network with people across the nation representing different institutions and hear about aspects of their work shedding light on the challenges they are facing at their respective institutions. Second, the workshops and working groups gave them a chance to deepen their relationships with their own champion team in a neutral environment that fostered trust and respect for diverse perspectives on community/patient engagement and institutional transformation. Third, participants valued the fact that not only was the patient voice invited to be part of the E2 training but that it was also listened to with the expectation that the ideas shared would be translated into *workable actionable items*. However, some participants remained skeptical about institutional willingness to be more responsive to the needs and demands of community members and patient advocates for equitable engagement in research because it required academics and institutional leaders to voluntarily share decision-making power over policies and resources. Finally, there remains doubt that the current usage of CBPR or CEnR to foster partnerships actually result in a win-win for both the institution and the community. The institution may have reaped what it set out to get from the relationship that informs their research. But it’s unclear that the community got a sustainable, funded working model to grow capacity, increase research knowledge, develop skill sets of its workers to engage, and be well prepared to join equally in the next project that comes its way.
